# Metformin in the management of antipsychotic-induced weight gain – why the ‘weight’?

**DOI:** 10.3389/fpsyt.2024.1491417

**Published:** 2024-11-12

**Authors:** Ita Fitzgerald, Laura J. Sahm, Ciara Ní Dhubhlaing, Sarah O’Dwyer, Jean O’Connell, Jennifer Torrens, Erin K. Crowley

**Affiliations:** ^1^ Pharmacy Department, St Patrick’s Mental Health Services, Dublin, Ireland; ^2^ Pharmaceutical Care Research Group, School of Pharmacy, University College Cork, Cork, Ireland; ^3^ Pharmacy Department, Mercy University Hospital, Cork, Ireland; ^4^ College of Mental Health Pharmacy, Burgess Hill, United Kingdom; ^5^ Department of Medicine, St Patrick’s Mental Health Services, Dublin, Ireland; ^6^ Centre for Obesity Management, St Columcille’s and St Vincent’s University Hospitals, Dublin, Ireland; ^7^ University College Dublin, Dublin, Ireland

**Keywords:** antipsychotics, weight gain, obesity, schizophrenia, psychosis, metformin

## Introduction

Antipsychotic-induced weight gain (AIWG) contributes significantly to the 2-3-fold higher rates of obesity seen amongst those living with a severe mental illness (SMI) (1). Clinically significant weight gain (≥7% increase in weight) has been linked to almost all antipsychotics (2). Adjunctive metformin treatment has been demonstrated to effectively reduce AIWG (3). However, metformin’s position within current AIWG management algorithms is typically limited to an option only to be considered after alternative interventions have been trialled and deemed unsuccessful (4–6). One of the most recent guidelines influencing the management of AIWG in psychiatry is the 2018 World Health Organisation (WHO) guideline on the management of physical health conditions in adults with severe mental disorders (4). Within this guideline, they recommend “*where lifestyle interventions and/or switching psychotropic medication do not appear successful, adjunctive metformin may be considered. This should be considered under close clinical supervision and monitoring”* (4). Leaving aside evidence addressing the comparative efficacy of pharmacological and non-pharmacological management interventions, whether such recommendations, and other similar guideline recommendations (5, 6), reflect the preferences of patients burdened with managing both a SMI and AIWG has recently been scrutinised within critiques of available management guidance (7, 8). Within this paper, we argue for much earlier and broader use of metformin to manage AIWG.

Evidence demonstrating the efficacy of metformin primarily as an early intervention agent, rather than - as conventionally thought - a treatment of established AIWG will be outlined. We aim to demonstrate how inadequate consideration of the typical trajectory of weight gain following antipsychotic initiation among susceptible patients, and associated timing of metformin introduction within trials assessing its efficacy, have led to inaccurate conclusions regarding its role within management algorithms. Frequent barriers to the use of metformin cited by psychiatric clinicians include concern regarding it’s risks and the appropriateness of prescribing glucose-lowering medications among cohorts without diabetes (9, 10). We will provide an overview of current risk estimates relating to established, but low risks of vitamin B12 deficiency and lactic acidosis. The rationale for why hypoglycaemia is not a concern when metformin is used in AIWG management will be discussed. Finally, the relationship between metformin and renal functioning and management strategies of common gastrointestinal side effects will also be discussed.

## Use of metformin to manage AIWG – by whom should we be guided?

Healthcare professionals can (often unconsciously) endorse stereotypical assumptions and stigmatising attitudes about those living with obesity (7, 11). Obesity management among those with a SMI may be particularly vulnerable to such implicit biases, where the experience of a ‘dual stigma’ has been described by those living with both obesity and a SMI (12). Recommendations addressing weight management in this cohort may be uniquely susceptible to ideology and sociocultural values regarding the appropriateness of anti-obesity medications and expectations of self-management (7). To date, recommendations addressing weight management within SMI cohorts have largely been informed by the expert opinion of guideline development groups - with no or minimal patient input (4–6). Until recently (7), availability of empirical evidence outlining patient values and preferences for management interventions, including their acceptability and transferability across cohorts and contexts, was relatively unexplored. The prognosis of AIWG is highly variable (13, 14). Thus, patient management preferences are likely also extensively variable. We believe the absence of this diverse lived experience representation within guideline development groups limits the applicability and clinical utility of current guideline recommendations, where the role of metformin remains inappropriately limited.

## Prognosis and aetiology of weight gain induced by antipsychotic treatment

The largest proportion of total weight gained occurs within the first 12 months of antipsychotic treatment (13). Whilst AIWG can continue after this time, this is typically at a much slower rate (13, 15, 16). In this way, the relationship between duration of antipsychotic treatment and weight gain can be described as hyperbolic (weight increases steadily early in treatment until a plateau is approached). This has been demonstrated with antipsychotics associated with medium- and/or high-risk of inducing clinically significant AIWG and is largely independent of dose (17, 18). Current understanding of mechanisms behind this hyperbolic relationship and eventual reaching of AIWG plateau suggests that antipsychotics interfere with homeostatic mechanisms regulating weight and total body fat mass, culminating in the development of a new body weight set-point (19). Mechanisms responsible for the orexigenic effects of antipsychotics are thought to be primarily attributable to their affinity to bind a broad range of neurotransmitter receptors in the central nervous system (CNS) (20). Through antagonism of specific subtypes of serotonergic, histaminergic, muscarinic, and dopaminergic receptors, antipsychotics interfere with the regulation of metabolic signals that communicate energy status and suppress appetite when the body has met its energy requirements (21, 22).

As the primary regulator of food intake and body weight, the hypothalamus regulates energy intake and expenditure via signalling mediated by neuromodulators, including the anorexigenic neuropeptides proopiomelanocortin (POMC) and cocaine and amphetamine-regulated transcript (CART) and the orexigenic neuropeptides, neuropeptide Y (NPY) and agouti-related peptide (AgRP). Antagonism of Histamine 1 (H1), 5HT (serotonin) 1A, 2A and 2C and Muscarinic 3 (M3) receptors by antipsychotics enhances appetite and reduces satiety through a final common pathway leading to up-regulation of the NPY-AgRP signals and downregulation of POMC. Antagonism of Dopamine 2 (D2) receptors in the mesolimbic pathway may lead to dysregulated eating by opposing the effects of pre-synaptic dopamine release within neurons connecting the ventral tegmental area to the *nucleus accumbens* involved in mediating satiety following food intake (20–22). The reward deficiency hypothesis posits that decreased dopaminergic signalling within the striatum may impair the reward response following food intake, resulting in compensatory increase in food cravings and excessive caloric intake (22).

However, antipsychotics with selective activity at D2 receptors through partial agonism (e.g., aripiprazole) or antagonism (e.g., amisulpride) are uncommonly associated with clinically significant AIWG (23). Additionally clozapine, often demonstrated as being the highest risk antipsychotic with regards to inducing AIWG (23), possesses little affinity for the D2 receptor (24). A high affinity for 5HT2A, 5HT2C and H1 appears important in mediating the most clinically significant presentations of AIWG (22). A more detailed discussion on current understanding of the aetiology of AIWG can be found elsewhere (20, 21). Understanding the aetiology of AIWG provides biological plausibility for the demonstrated benefit of metformin in preventing further weight gain when introduced early in antipsychotic treatment, i.e., before a plateau of AIWG is reached.

## Metformin interference with mechanisms mediating AIWG

The capacity of metformin to attenuate AIWG lies in its ability to oppose both hyperphagia and reduced satiety induced by antipsychotic treatment. The mechanisms by which metformin exerts anorexigenic effects are still emerging, with evidence of direct and indirect effects on the gastrointestinal tract, the gut microbiome, the CNS, and the gut-brain axis (25).Although peripheral activation of AMP-activated protein kinase (AMPK) is a major pathway for the metabolic benefits of metformin, this effect appears to be tissue-specific. Within the hypothalamic appetite regulatory centres, metformin has been shown to inhibit AMPK and decrease orexigenic NPY expression (26). Within the gastrointestinal tract, metformin has been shown to increase secretion of the gut-derived anorectic hormones, glucagon-like peptide 1 (GLP-1) and peptide YY by enteroendocrine L cells via activation of intestinal 5’AMPK-dependent pathways and alteration of bile acid absorption (25, 27). Within animal models, treatment with metformin is associated with an increase in leptin receptor expression and decrease in hypothalamic leptin resistance (28). A more detailed overview of the suggested mechanisms through which metformin decreases total caloric intake is beyond the scope of this paper, and can be found elsewhere (25). Although traditionally the benefit of metformin has been assessed from the perspective of absolute weight reduction (3, 29), use of pharmacological interventions in inducing feelings of satiety is an additional, patient-reported outcome demonstrated as important among those prescribed antipsychotics (7), but is rarely considered in discussions about the role of pharmacological adjuncts in managing AIWG (3, 29).

## Metformin – health gains versus weight loss

The primary rationale for delayed introduction of metformin within AIWG treatment guidelines is limited efficacy in reversing the typically significant burden of weight gain induced by antipsychotic treatment. A measure of 2-3kg weight reversal is often cited (4–6). This “modest” weight reduction, alongside consideration of risk of side effects, increased tablet burden and concerns about adherence, are all arguments used to support delayed introduction of metformin (5, 6). Efficacy of metformin in reversing AIWG is, however, often presented with an implicit assumption that a plateau of weight gain has occurred. To draw accurate conclusions regarding the efficacy of any intervention in managing AIWG, the duration of antipsychotic treatment and prognosis of AIWG must be considered, specifically whether continued weight gain is likely.

Metformin treatment has been demonstrated to be more effective when prescribed in the context of first episode psychosis (FEP) (10, 29). Compared to those with an established psychotic illness, those with FEP likely have minimal, or no, prior antipsychotic exposure. In one example, a subgroup analysis of a systematic review tested the hypothesis of differential efficacy among those with varying illness chronicity (29). Metformin was more efficacious among those experiencing FEP compared to those with so-called ‘chronic psychosis’ i.e., extensive prior antipsychotic treatment. Among those treated with metformin, mean weight reduction was significantly higher among the FEP cohort, mean difference -3.24kg, 95% CI [-4.55 – (-1.92)], p <0.001. On closer inspection of individual studies included in the analysis, weight loss in both metformin-treated groups was comparatively similar, with median weight reductions of – 2.37kg in the FEP cohort, and – 1.56kg in the chronic psychosis groups. Rather, observed differences between the FEP and chronic psychosis groups are largely accounted for through dissimilarities in the weight gain trajectory of the placebo-treated groups of both cohorts. Corresponding median weight increases over an average of 16 weeks in the antipsychotic-placebo-treated groups were 2.5kg (FEP) and 0.16kg (chronic psychosis) (29). Median measurements are cited here as a more accurate representation of central tendency, given the interindividual variation in AIWG trajectory.

The primary benefit of metformin as a weight control agent in preventing further or inducing modest weight reductions can also be seen in more recent studies among those who are antipsychotic-naïve or initiating a new antipsychotic (30–32), and in studies including child and adolescent populations (33, 34). Metformin’s benefits have also been demonstrated to extend to those prescribed clozapine in the context of treatment-resistant schizophrenia (35, 36). Given the typical short length of randomised controlled trials (≤6 months) (29–34, 36), and recommended duration of antipsychotic treatment extending far beyond this (23), average weight change figures underestimate the impact of AIWG on final body weight, and thus, the benefit of early metformin initiation.

## Contextualising risk concerns

**Figure 1:**
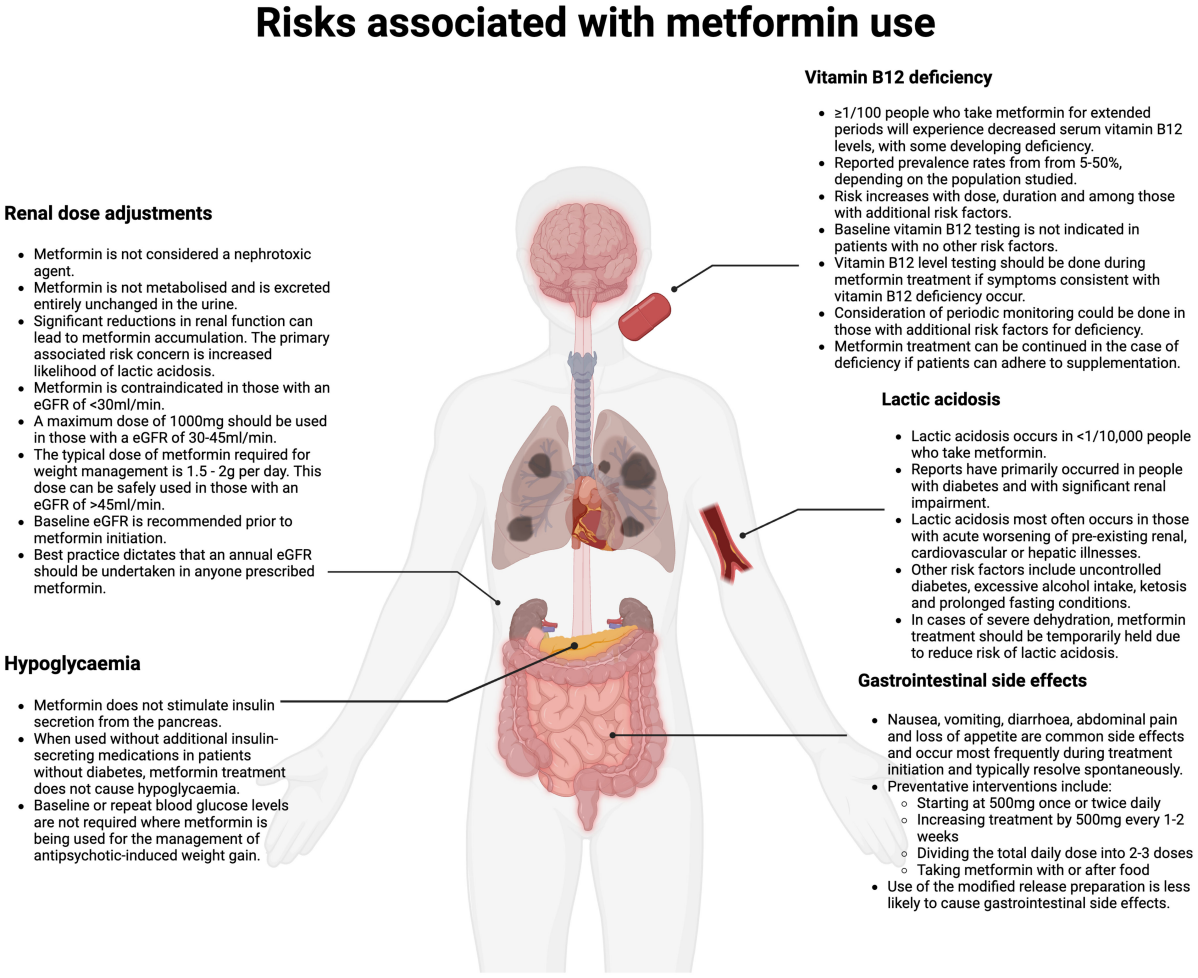
Risks associated with metformin prescribing (Created in BioRender. Fitzgerald, I. (2024) /BioRender.com/m67m178).

Concerns regarding risk of serious side effects have been reported as a barrier to use in settings outside of diabetes management (9). Serious side effects associated with metformin use include lactic acidosis and vitamin B12 deficiency. Potentially serious side-effects associated with metformin use include lactic acidosis and vitamin B12 deficiency. Metformin-induced lactic acidosis is rare, and clear recommendations exist regarding when to avoid prescribing in high-risk groups (37). Vitamin B12 deficiency can occur with continued use, although is manageable with appropriate monitoring and supplementation, where required. Gastrointestinal side-effects are common on metformin initiation, but are usually transient, and can be managed by a series of preventative measures outlined below. **Figure 1** gives an overview of the prevalence of such side-effects and management advice (8). Misconceptions about metformin being nephrotoxic may also serve as a barrier to more widespread use and are addressed in **Figure 1**.

## Discussion

Early initiation of metformin is essential for those taking antipsychotics to benefit from its weight stabilisation properties. Current AIWG management algorithms endorse limiting use of metformin to those for whom lifestyle interventions and/or switching antipsychotic appear ineffective (4–6). However, interpretations drawn from reviewing studies assessing the efficacy of metformin, without considering the timing of metformin initiation and typical prognosis of AIWG, substantially underestimate the benefit of metformin for most patients in managing AIWG. Thus, an unintended consequence of current guideline recommendations is delayed use of a pharmacological intervention that is largely effective in preventing further weight gain, not significantly reversing established AIWG (30). This understanding of metformin, in modifying the prognosis of AIWG when introduced early in antipsychotic treatment, has been largely unaccounted for within discussions first addressing metformin’s role in AIWG management published over 20 years ago (38).

For most, by the time metformin is started, the unique weight management benefits of this agent have been lost. Furthermore, compared to effective, resource-intensive dietary and exercise interventions (3), or use of costly GLP-1 agonists, metformin use in AIWG management represents an intervention associated with equitable implementation opportunities. Metformin is included on the WHO’s essential medication list (39), and is associated with minimal drug-drug interactions, including psychotropic medications (37). As such, with its favourable benefit-risk profile, metformin can be accessed across contexts and cohorts and is safe for initiation within psychiatric settings (8).

Further trials, similar to existing ones, are very unlikely to change our understanding of metformin’s role in managing AIWG. Larger, extended studies would be helpful in ascertaining whether benefits of earlier use of metformin, such as benefits in reducing cardiovascular risk demonstrated among those with diabetes in the UK Prospective Diabetes Study (40), are replicated within SMI cohorts without diabetes. Accumulation of safety data in diverse populations has demonstrated metformin to be associated with a very low absolute risk of serious side effects (37, 41). The reality of the current, and potentially worsening (1), burden of excess morbidity and mortality amongst those with a SMI also needs to be acknowledged when considering interventions to address AIWG (42).

## Implementing paradigm shifts in general obesity management into psychiatry

As in general overweight and obesity management, heterogeneity in response to any intervention is to be expected (43). Thus, a one-size-fits all management algorithm, currently endorsed within AIWG management guidance, does not reflect current understanding of the pathophysiology of obesity more generally (5, 6), nor indeed the preferences of patients with experience of managing AIWG (7). Thus, an individualised and more patient-centred approach to weight management in psychiatry is required. Accordingly, pharmacological adjuncts beyond metformin are needed for effective AIWG management. Should metformin fail to plateau, or meaningfully slow the trajectory of AIWG, prompt intervention is required to protect weight status. Evidence for the role of GLP-1 agonists in preventing and reversing established AIWG remains in its infancy (44, 45). Aside from novel treatment strategies, research using implementation science methods is also required to support effective design of psychiatric settings in delivering proactive weight management services. Availability of dedicated AIWG management guidance co-produced by endocrinology, psychiatric clinicians and patients with lived experience is one example of a targeted intervention that may increase uptake of evidence-based weight management practices within psychiatry (46).
